# A Role for Peroxisome Proliferator-Activated Receptors in the Immunopathology of Schistosomiasis?

**DOI:** 10.1155/2012/128068

**Published:** 2011-06-09

**Authors:** Barrie J. Anthony, Jeremy T. Allen, Yuesheng S. Li, Donald P. McManus

**Affiliations:** ^1^Queensland Institute of Medical Research, Post Office Royal Brisbane Hospital, QLD 4029, Australia; ^2^Department of Biology, Centre for Parasitology and Disease, Biomedical Sciences Research Institute, University of Salford, Manchester M5 4WT, UK

## Abstract

Peroxisome proliferator-activated receptors (PPARs) have been demonstrated to have a role in immune regulation. In general, they are anti-inflammatory and promote Th2 type responses, and they are associated with the alternative activation of macrophages. Interestingly, helminth infections, such as the schistosome blood flukes that cause schistosomiasis, are characterised by a Th2 response and the accumulation of alternative activated macrophages. This would suggest that at some level, PPARs could have a role in the modulation of the immune response in schistosomiasis. This paper discusses possible areas where PPARs could have a role in this disease.

## 1. Introduction

The peroxisome proliferator-activated receptors (PPARs) are a group within the 48 transcription factors of the nuclear hormone receptor family involved in lipid metabolism and inflammation [1]. To be transcriptionally active, they require hetrodimerisation with the retinoid X receptor (RXR) to which the resulting heterodimers bind with peroxisome proliferator-response elements (PPREs) on DNA after activation by a ligand-to-modulate transcription [2]. The PPREs are located at the 5′ end of the target gene and consist of a repeat sequence—AGGTCA—separated by one nucleotide [3]. The binding to the PPRE is orientated with PPAR at the 5′ end and RXR towards the 3′ end [2]. For transcriptional control to occur, the PPAR/RXR heterodimers have to interact with coactivators or suppressors for stimulation or inhibition of target-gene expression, respectively [2]. The PPARs can also block transcription of other genes by interacting with other transcription factors by non-genomic transrepression, whereby they inhibit transcription by preventing dissociation of corepressors or sequester co-activators needed for binding of the transcription factor to the DNA [4]. There are 3 isoforms of the PPAR receptors, PPAR*α*, PPAR*β*/*δ*, and PPAR*γ* [5]. PPAR*α* is expressed in the liver, brown fat, heart, and skeletal muscle which have high levels of fatty acid catabolism, while PPAR*γ* is expressed in adipose tissue, the colon, and in macrophages, it is the major regulator in adipocyte differentiation and is a determinant in insulin sensitivity [3]. PPAR*β*/*δ* is ubiquitously expressed and is thought to have a role in metabolic disorders [3]. Polyunsaturated fatty acids and eicosanoids act as natural ligands for these receptors; however, synthetic ligands exist such as fibrates that target PPAR*α* and the thiazolidinediones that target PPAR*γ* [3].

PPARS have been demonstrated to be important in a number of different disease states such as metabolic disorders [3], inflammation [6], malaria [7], Chagas disease [8], and leishmaniasis [9]. Recent studies have revealed a role for PPARs in the control of the immune response. In general, they are anti-inflammatory [10], promote the development of alternatively activated macrophages (AAMΦ) [11], and are Th2 biasing [12]. Helminth worms have an incredible ability to modulate the host immune response and, in general, promote a Th2-biased environment that commonly involves the generation of AAMΦs [13], as occurs during schistosome infection. The fact that this parasite and other helminths induce Th2 biasing, with accumulation of AAMΦs, suggests that at some level, PPARs could be involved. This paper will explore the current state of knowledge in this area, focusing on the role of PPARs in the immunopathology of schistosomiasis and their potential as novel therapeutic targets.

## 2. Immune Regulation in Schistosomiasis

Schistosomiasis is a major health problem responsible for significant morbidity and mortality worldwide. It is estimated that approximately 200 million people are infected, causing severe disease in 20 million people [14]. The disease is caused by infection with the trematode worms, the schistosomes, of which *Schistosoma mansoni*, *S. japonicum*, and *S. haematobium* are the most important in regards to human disease [15]. Pathology is associated with the host's immune response to the eggs which results in a granulomatous reaction [16]. Helminth parasites are able to modulate the host immune response, allowing them to have good longevity within the mammalian host. Helminth infections are noted for polarising the immune response more towards a Th2 response characterised by interleukin (IL)-4, 5, and 13, large amounts of IgE, and by CD4^+^ T cells [17]. In schistosomiasis, the immune response is characterised by a switch from an early proinflammatory Th1 response to a Th2 response to eggs released by the female worm [18]. The Th2 response has the characteristics described above and is associated with AAMΦs ([19–21]). Cytokines such as INF-*γ*, IL-2, IL-12, and TNF-*α* are associated with the early Th1 response and are repressed during this switch [18]. Helminth parasites can achieve this modulation the by release of soluble factors which can interact with host immune cells [13]. There is good evidence for this in schistosome infections in which both live or dead eggs injected into naive mice rapidly induce a Th2 response [20]. Egg-derived products have been observed to drive the switch from the Th1 response to the Th2 response. Examples of this are the IL-4-inducing principal of *S. mansoni* eggs (IPSEs) that is secreted from the egg subshell into the surrounding granuloma area and has been demonstrated to induce human basophils to produce IL-4 and IL-13 [22]. Similarly, the glycoprotein, omega-1 that is secreted by *S. mansoni* eggs and present in secreted egg antigen (SEA), has been observed to drive human monocyte-derived dendritic cells towards Th2 polarisation and to generate Th2 responses *in vivo* in mice [23]. Egg-derived glycoconjugates, *α*3-fucosyltransferase, and core g 2-xylosyltransferase have been used with dendritic cells to produce a Th2 response in the murine model of disease caused by *S. mansoni* [24].

## 3. Immune Regulation by PPARs

It is believed that PPARs may be important in the regulation of the immune response, a role supported by the fact that PPARs have been described in monocytes, macrophages, neutrophils, peripheral blood lymphocytes, T cells, B cells, natural killer cells, dendritic cells, eosinophils, and mast cells [1]. Supporting this, ligands of PPARs have been shown to have a therapeutic role in several models of inflammatory and autoimmune diseases [6]. PPAR*γ* agonists have been demonstrated to have anti-inflammatory effects in renal injury [25], murine carotid atherosclerosis [26], and in oxidative stress induced in a human diploid fibroblast model of aging [27]. PPAR*β*/*δ* agonists have additionally been demonstrated to have a protective role in a murine model of autoimmune encephalomyelitis [28]. Further, PPAR*γ* agonists have been observed to inhibit the production of TNF-*α* in human monocytes [10].

Part of the anti-inflammatory mode of action of PPARs is due to the fact they can interact with transcription factors involved in inflammation such as NF-*κ*B, activator protein-1 (AP-1), and signal transducers and activators of transcription (STAT) at a transcriptional level ([29, 30]). In the case of NF-*κ*B and AP-1, PPAR*α* has been shown to interact directly with p65, c-Jun, and CBP, thereby interfering with their transcriptional capacity [29], while the PPAR*γ* agonist, 15d-PGJ2, inhibits STAT signalling indirectly [30]. Interference of these pathways results in the downregulation of the Th1 proinflammatory cytokines TNF-*α*, IL-1, -6, and 12 ([29, 30]).

**Figure 1:**
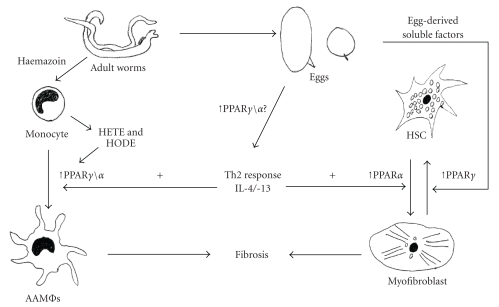
Summary of possible interactions in schistosomiasis with PPARs. This figure displays the possible pathways in which PPARs could be involved in schistosomiasis. PPARs could be involved in immune regulation, as they are associated in generation of a Th2 response. PPAR*βγ*/*α* both cause downregulation of Th1 cytokines and promote IL-4/-13 production. PPARs have a role in the alternate activation of macrophages where PPAR*γ*/*α* have been demonstrated to be essential for this process. In schistosomiasis, AAMΦs have a protective effect and a role in Th2 biasing. Schistosomes could interact with this activation indirectly via induction of IL-4/-13 production and directly by the breakdown products of hemozoin, which can interact with PPAR*γ*/*α*. In terms of pathology, the PPARs could interact with the transdifferentiation process of HSCs into fibrogenic myofibroblasts. They could limit this process by inhibiting transdifferentiation associated with increased PPAR*γ*, whereas PPAR*α* would be associated with generation of the fibrogenic myofibroblast.

Notably, PPARs have been demonstrated to result in upregulation of Th-2 responses and downregulation of Th-1 responses. An agonist of PPAR*α*, gemfibrozil, results in increased number of GATA3 positive T cells in the spleens of donor mice as well as the stimulation of its expression and DNA-binding activity resulting in IL-4 production [12]. In the same study, gemfibrozil was additionally observed to inhibit the expression and DNA-binding activity of T-bet, causing a decrease in INF-*γ* production. IL-4 can interact with PPAR*γ* indirectly and directly in macrophages [31]. PPAR*γ* expression is both directly and indirectly upregulated by IL-4. IL-4 will induce target-gene expression by increasing PPAR*γ* expression and by increasing the production of PPAR*γ* ligands via 15-lipoxygenase, which results in lipoperoxidation products such as linoleic acid (HODE) or arachidonic acid (HETE) [31]. Schistosomes and other helminths could potentially interact with PPARs through these pathways. They could do this indirectly via IL-4 and IL-13 as both cytokines can activate PPAR*γ* resulting in the suppression of the proinflammatory response and activation of AAMΦs which favour the establishment of a chronic parasite infection [1]. Schistosomes could potentially interact with PPARs via hemozoin. Schistosomes produce hemozoin as a product from feeding on mammalian host red blood cells; its structure is identical to malarial hemozoin [1]. It is composed of a complex mixture of neutral lipids and polyunsaturated lipids from which lipoperoxidation products HETE and HODE acid, which are natural ligands for PPAR [1], are derived (Figure 1). Carter et al. [32] showed that macrophages that have previously phagocytosed schistosomal-derived hemozoin have a reduced ability to produce iNOS in response to LPS.

## 4. PPARs and Alternatively Activated Macrophages

Macrophages have multiple roles with regards to the host immune response. They have a role in early detection of invading pathogens, both as antigen presenting cells (APCs) that initiate a host response and as effecter cells that can act to kill the invading pathogen [33]. Macrophages used to be classified as either activated or deactivated, but in recent years, this has changed to classically activated macrophages (CAMΦ) and AAMΦs [33]. CAMΦs are induced by INF-*γ*, TNF-*α*, and LPS and produce proinflammatory cytokines such as IL-1*β*, IL-12, IL-23, and TNF-*α* and the chemokines CXCL-9, -10, -11, and -16 [33]. AAMΦs are associated with production of IL-10 and are induced by IL-4 and IL-13 [33]. This classification has been expanded in recent years with proinflammatory macrophages being termed M1, while anti-inflammatory macrophages are termed M2. The M2 macrophages have been divided into different subsets, whereby AAMΦs are classified as M2a cells which are defined by low expression levels of IL-12 [34], or are M2b cells, which release high levels of IL-10 on activation by immune complexes, and M2c cells which are induced by IL-10 and are believed to be more similar to CAMΦ [33]. One of the main differences distinguishing AAMΦs from CAMΦs is in how they metabolise L-arginine. CAMΦ metabolise L-arginine into NO via iNOS, while AAMΦ metabolise L-arginine into urea and L-ornithine via arginase-1 (arg-1) [35].

AAMΦ have been associated with many helminth infections with many different roles attributed to them. In infection with the nematode *Brugia malayi*, AAMΦs are associated with Th-2 biasing [36, 37], while in infection with *Heligmosomoides polygyrus*, AAMΦs have a role in parasite clearance and host protection [38]. In cestode infections, AAMΦs have been associated with downregulation of the immune response in *Echinococcus multilocularis* [39] and Th-2 biasing in *Taenia crassiceps* infection as well as favouring parasite survival [40]. In *Schistosoma* infection, they have a role in Th-2 biasing as well as in downregulation of the Th-1 response and mediate immunopathology promoting host protection, but, at the same time, they promote progressive pathology due to granuloma formation ([21, 41]). Overall, their role seems to be host protective by causing downregulation of overaggressive inflammatory reactions, but they are also protective for the parasite, forming part of the immunomodulation strategy needed for successful colonisation of the host.

PPARs could potentially have a role in helminth infections by regulating AAMΦs. There are a number of studies demonstrating PPAR*γ* as essential for AAMΦ activation and maturation in other disease states such as metabolic syndrome and leishmaniasis ([9, 11]). In metabolic syndrome, it has been shown with macrophage-specific PPAR-*γ* knockout mice that PPAR*γ* is essential for AAMΦ maturation resulting in the mice developing diet-induced obesity, insulin resistance, and glucose intolerance [11]. Additionally, PPAR*γ* and PPAR*δ* agonists have been observed to mediate arginase-1 expression in macrophages, and this expression is blocked in macrophages from PPAR*γ*- and PPAR*δ*-deficient mice [11]. Interactions between IL-4/13 and PPARs have been extensively studied in leishmaniasis in which PPARs promote AAMΦ-mediated susceptibility to the disease by stimulating intracellular amastigote growth in infected macrophages [11]. This is due to the lack of NO production in resultant AAMΦ which is essential for amastigote killing.

## 5. DO PPARs Modulate Host Pathology?

The hepatic stellate cell (HSC) is located within the liver sinusoid in the space of Disse where it is responsible for storage of vitamin A and the maintenance of a low-density matrix between the liver endothelium cells and the hepatocytes [42]. Maintenance of this matrix is important as it allows solutes in the plasma to reach the hepatocytes unimpeded, allowing the liver to function correctly [43]. In response to insult or injury to the liver, HSCs can undergo a process of transdifferentiation from the quiescent vitamin A-storing cell to a myofibroblast responsible for the accumulation of scar tissue within the space of Disse [44]. This has highlighted parallels between this cell type and that of the adipocyte which can undergo a similar process [45]. Adipocytes differentiate from a fibroblast-like preadipocyte and become lipid laden associated with the expression of PPAR*γ* [46]. Quiescent HSCs express PPAR*γ* which upon transdifferentiation into a myofibroblast-like cell lose their ability to store lipid droplets as the expression and activity of PPAR*γ* decrease [47]. This has suggested a role for PPAR*γ* agonists in the treatment of fibrosis. PPAR*γ* agonists have been demonstrated to cause reversion of the myofibroblast back into a quiescent HSC ([45, 47]).

Recent studies have implicated a role for HSCs in the pathogenesis of schistosomiasis [48]. Activated HSCs have been observed in the murine model of disease and at the end stage of human disease with *S. japonicum* [49] and human disease with* S. mansoni* [50]. Notably, the PPAR*γ* agonist rosiglitazone has been demonstrated to prevent fibrosis in *S. japonicum* infection of mice [51]. In this study, mice cotreated with the antischistosome drug praziquantel and rosiglitazone induced reduced expression of collagen 1 and 3, *α* smooth muscle actin (a marker for myofibroblasts), inflammation, increased expression of PPAR*γ*, reduced NF-*κ*B-binding activity, and reduced TNF-*α* levels [51]. In a recent study, Anthony et al. [52] showed that eggs of *S. mansoni* could downregulate fibrogenesis in the human HSC cell line, LX-2, causing regression from the activated myofibroblast to the quiescent HSC. This downmodulation was associated with increased expression of PPAR*γ* at the gene level as well as with the accumulation of lipid droplets within the cytoplasm of HSCs. At the granuloma level, fibrosis first occurs towards the periphery of the granuloma site, and it was postulated in this study that antigens from the egg may inhibit fibrosis in close proximity to the egg as it is not until the egg is killed and destroyed that fibrosis occurs throughout the granuloma area. However, PPAR*γ* could act as a double-edged sword, as it would be involved in alternative activation of macrophages at the granuloma site, which in turn can be responsible for collagen production by the production of arginase-1 which promotes the production of proline. Additionally, the Th2 response is profibrogenic and high levels of IL-13 are associated with fibrosis in schistosomiasis [53].

## 6. Conclusions

Schistosomiasis is characterised by a switch from an early Th1 response to a Th2 response and accumulation of AAMΦs in response to eggs released by the schistosome worms. PPARs have been demonstrated to cause downregulation of proinflammatory Th1 cytokines while simultaneously upregulating Th2 responses. They have additionally been shown to be essential in the alternative activation of macrophages. This suggests that PPARs may play a role in the regulation of the host response to schistosome antigens. Additionally, it has been demonstrated that *S. mansoni* eggs cause downregulation of fibrogenesis in the human-derived HSC cell line, LX2, a response associated with increased expression of PPAR*γ*, and accumulation of lipid droplets within the cell's cytoplasm. Rosiglitazone, a PPAR*γ* ligand, has been additionally been demonstrated to reduce pathology associated with *S. japonicum* infection in mice. The possible interactions with schistosomiasis and PPARs are summarised in Figure 1. Further studies of the role of PPARs in this disease and those caused by other helminth infections are, therefore, warranted and may help in the identification of new antipathology drug and vaccine targets for schistosomiasis and other important diseases caused by the parasitic helminths.

##  Conflict of Interests

The authors declare that they have no Conflict of interests.
